# Annotations

**Published:** 1906-10-27

**Authors:** 


### ANNOTATIONS.

### The Taxation of Motor-Cars.

If the proposals of the Committee appointed to inquire into the Motor Car Act are carried out, the taxation of cars under 1 ton will be materially increased. For instance, cars over 12 cwt., but under 15 cwt., are to pay three guineas per annum; those over 15 cwt., but under 25 cwt., are to pay five guineas. This will in many cases be a great hardship to medical men. In order to ensure protection from the weather in winter time, it is necessary to have a covered-in body, this, again, means a heavier frame, and also a stronger engine to get the extra weight uphill. A car of this kind cannot be built to last, under 18 to 20 cwt. Medical men will, therefore, be called upon to pay five guineas a year instead of two guineas as at present. We think this is very unfair, unless the doctor's car is classified as a trade vehicle, for which half the above rate is recommended. Even then there will be an increase of 20 per cent. on the present tax. The Committee in their report appear to have a strong objection to basing the taxation on horse-power on account of the difficulty of estimating the power of the engine. This opinion is erroneous. During the past season competitions have been constantly taking place by handicap based on the difference in horse-power of various engines. This difference has been worked out in a few minutes by amateur umpires on the basis of the Automobile Club formula, and everybody has been satisfied. If the Legislature wish to increase the burden of taxation for the rich man and the road hog, it must base its proposals on horse-power instead of weight.

### The Street Ambulance in London.

A statement explaining the present position of the street ambulance movement has been issued by Mr. Reginald Harrison. The claim for a more efficient ambulance service in London is now generally recognised, and more especially by those who are familiar with the arrangements that have long existed in other large towns and cities. Agitation has been efficiently, if quietly, conducted, so that the whole matter is now in the hands of the Home Secretary, from whom an efficient and reasonable settlement may be confidently expected. This settlement must satisfy certain conditions on which all interested are practically agreed. First it must provide an improvement in the mere mode of conveyance, for it is not more than the truth that, as stated by Sir Cooper Perry, "it is very painful to watch the arrival of accidents at hospitals under the present system." Secondly, no system can be satisfactory which does not include rapid transit vehicles—horse or motor power—by means of which injured persons can be carefully and rapidly conveyed to hospitals, or to their own homes, as the case may be; with the ambulance should be provided the services of a competent "first-aid" attendant. Another important consideration is the advisability of utilising and keeping in touch with existing organisations, and this partly in recognition of services already rendered, and partly for the sake of avoiding unnecessary expense. This latter aspect of the question is not unimportant, but it will universally be allowed to be a secondary consideration. The increasing complexity of the street traffic in London renders a certain number of accidents unavoidable, and it is not short of a scandal that even one of the unhappy victims of these should be left without efficient succour in the richest city in the world. Imperfect help in such cases is not merely a negative defect. In addition, it often means an aggravation of the effects of the accident, and an added risk to the patient's life.

### Counter-Prescribing.

Our excellent contemporary the *Pharmaceutical Journal* seems forced at occasional intervals to find some kind of excuse or explanation for what it calls the "counter-practice" of the prescribing chemist. Its latest suggestion is that some essential difference exists between this habit or custom and the "dangerous pretensions of irresponsible persons who profess to diagnose diseases." Yet it defends the treatment by a chemist of a discharge from the nostrils or an attack of diarrhœa on the ground that "an intelligent person with no medical training" is generally able to judge quite as well as a medical man whether such symptoms are of trivial import or whether they mean serious organic disease—a proceeding which it is difficult to distinguish from the diagnosis of disease by irresponsible persons. This particular position our contemporary endeavours to strengthen by the oracular announcement that it is only "as a rule" that clinical training must precede diagnostic skill, there being presumably numerous exceptions to this demand among the ranks of the chemists and druggists. The contention that the prescribing druggist should be placed in a different position from other "irresponsible persons who profess to diagnose diseases" cannot for a moment be admitted. Upon one and all rests the charge that they assume functions for which they have not been trained. That in many individual instances the risk to the sick man involved by all this amateur prescribing is small, is true, but it is equally true that now and again the consequences are disastrous. If members of the public like to accept advice regarding their health from the chemist they are as free to do so as they are to take advice from any other unqualified person. The criticism upon persons adopting such a policy refers not to their right but to their wisdom. And the reason why the pharmacist is often specially reflected on is not that he poaches on "the preserves of the medical profession," but because having received some measue of scientific training better things might have been expected from him. If, however, the counter-prescriber and his customers are satisfied to accept the risk, such as it is, the affair is for themselves to decide. Only the principal performer must not hope to escape the label which he endeavours to attach to the pretensions of his rivals.

